# Triple Arthrodesis for Adult-Acquired Flatfoot Deformity

**DOI:** 10.1177/2473011419849609

**Published:** 2019-08-19

**Authors:** MAJ Uma E. Erard, MAJ Andrew J. Sheean, Bruce J. Sangeorzan

**Affiliations:** 1 Department of Orthopaedic Surgery, San Antonio Military Medical Center, San Antonio, TX, USA; 2 Orthopedics and Sports Medicine, University of Washington, Seattle, WA, USA

**Keywords:** arthrodesis, triple, planovalgus, deformity, arthritis

## Abstract

Originally described as a means to address fixed deformities or uncontrolled movement of the hindfoot observed in paralytic foot deformities, triple arthrodesis has evolved into a powerful procedure for the correction of a variety of foot deformities. Over the past decade, multiple advances have been made with respect to diagnostic imaging, fixation options, bone graft substitutes, and postoperative regimens. While this operation requires experience and skill to execute, when properly performed, it allows for correction of deformity and a plantigrade and ideally pain-free foot for ambulation. The purpose of this review is to highlight advances in the procedure and its application to the rigid planovalgus foot.

**Level of Evidence:**

Level V, review.

## Background and Historical Perspective

**Figure 1.**
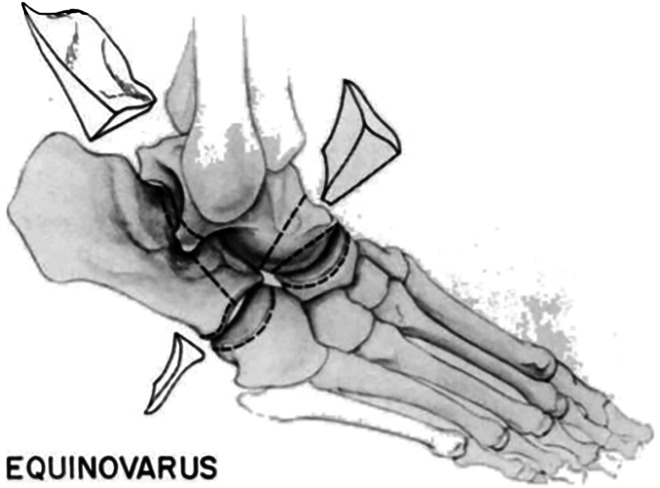
Hoke triple arthrodesis.

Triple arthrodesis is a powerful tool for the treatment of foot deformity as it allows for correction in the coronal, sagittal, and axial planes. In the patient with neuromuscular disease, triple arthrodesis offers a means of deformity correction and stabilization without the risk of recurrence carried with isolated soft tissue and periarticular procedures. In the patient with arthrosis and deformity, it offers pain relief and correction. The advent and widespread use of internal fixation in the foot allows for the rigid fixation of multiple joints and obviates the necessity for external hardware and/or prolonged casting.^70^ The origins of the triple arthrodesis date back to the early 1900s when the procedure was aimed at treatment of a variety of conditions ranging from idiopathic pes cavus and planus to deformities related to paralytic conditions of the foot. In 1908, Royal Whitman devised a surgery to address calcaneus and calcaneovalgus deformity due to neuromuscular abnormalities. Previously described is removal of the talus and backward displacement of the foot but noted this was not as successful for equinovarus deformities as it was for calcaneovalgus deformity.^85^ The Hoke triple arthrodesis was used for deformity correction in the days before internal fixation and changed the foot position by removing a portion of the talus and reshaping the residual talus followed by fusion of the subtalar and calcaneocuboid joints (Figure 1).^38^ Another study later reviewed patients undergoing this procedure and found a 6.5% rate of osteonecrosis of the talus when the talar head resection was performed proximal to the origin of the artery of the tarsal canal.^28^ Hoke’s technique was then modified such that talus resection was performed distal to the artery, which resulted in elimination of the observed rates of talus osteonecrosis. Triple arthrodesis became the favored approach over isolated tendon transfers in the treatment of paralytic foot deformities related to poliomyelitis. Unfortunately, recurrence rates as high as 19% were observed. The addition of an osseous correction proved an exciting addition, and despite early treatment failures, surgeons accepted the procedure as an effective means of achieving and maintaining a pain-free, plantigrade foot.^11,81^ The current use of triple arthrodesis ranges from salvage procedures for rigid and spastic conditions in the pediatric and adult population to posttraumatic arthritic conditions, diabetic foot deformity, and rigid adult cavovarus and planovalgus deformities.^19,67^

## Biomechanics and Staging of the Rigid Flatfoot

Throughout the phases of the gait, the foot undergoes complex 3-dimensional changes through the hindfoot and transverse tarsal joints to allow for supple accommodation to the ground followed by rigidity for push-off. There are multiple theories regarding the etiology of this particular disorder, including primary posterior tibial dysfunction, spring ligament pathology, medial column hypermobility, and gastrocnemius or tendoachilles contracture as the driving force. The development of a rigid pes planovalgus foot is likely multifactorial in nature, involving a combination of the above disorders.^63^

A number of staging systems can be used to guide decision making for the operative treatment of pes planovalgus. Initially, it was classified into three stages depending on severity and rigidity.^42^ Later, a fourth stage was added to describe pathology at the ankle (Table 1).^21,58^ While there is some variability in the procedures recommended for the treatment of the flexible flatfoot, it is universally agreed that arthrodesis is the preferred option for treating the rigid flatfoot. Other indications for triple arthrodesis include posttraumatic arthritis of the triple joint complex, spastic and paralytic conditions, loss of soft tissue constraints causing atraumatic subtalar dislocation, and severe tarsal coalitions.

**Table 1.** Classification of Adult-Acquired Flatfoot Deformity.

Stage	Deformity	Operative Management^a^
I	No deformity	Tenosynovectomy, tendon transfer, MDCO
IIa	Mild/moderate deformity, <30% talar head uncoverage	Tendon transfer, MDCO, Cotton osteotomy
IIb	Severe flexible deformity, >30% talar head uncoverage	Tendon transfer, MDCO, lateral column lengthening, Cotton osteotomy vs first TMT fusion, triple arthrodesis
III	Fixed deformity of the triple joint complex	Triple arthrodesis
IV	Foot deformity with ankle deformity (lateral talar tilt)	Correction of foot deformity, deltoid reconstruction, ankle arthrodesis vs arthroplasty
IVa	Foot deformity is flexible	Correct foot deformity as outlined for IIb, correct ankle deformity as stage IV above
IVb	Foot deformity is rigid	Correct foot deformity as outlined for stage III, correct ankle deformity as stage IV above

Abbreviations: MDCO, medial displacement calcaneal osteotomy; TMT, tarsometatarsal.

^a^Nonsurgical modalities should be considered first.

## Clinical Evaluation

A focused history and physical should be obtained to include quality and duration of symptoms as well as relieving and exacerbating factors. Evaluation of the patient’s current function and functional goals is also important. Risk factors for nonunion should be identified for operative planning and counseling purposes. Tobacco use, specifically cigarette smoking, creates a relative risk of nonunion 2.7 times higher than that of nonsmokers.^40^ Preoperative anemia was also found to be associated with an increased complication rate and length of hospital stay in anemic patients undergoing hindfoot and ankle arthrodesis.^26^ Optimizing nutrition and long-term blood glucose control is also essential to decrease the chance of postoperative soft tissue and osseous complications. Neuropathy, regardless of its etiology, and a HbA1c >8 mg/dL are both independently associated with surgical site infection.^87^ Vitamin D levels should also be optimized. One recent retrospective study found that patients with vitamin D deficiency were 8.1 times more likely to develop nonunion after elective foot and ankle reconstruction.^56^

**Figure 2.**
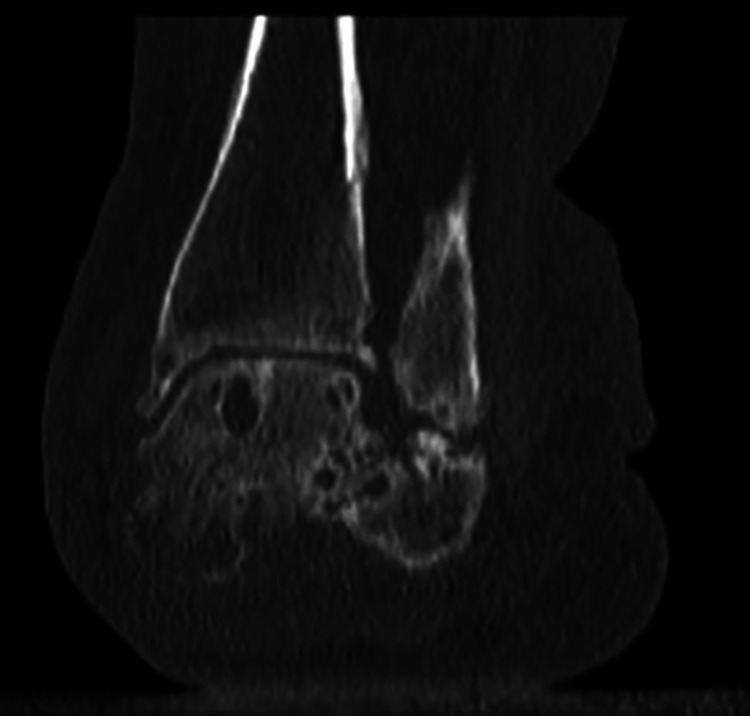
Computed tomography scan of severe hindfoot valgus resulting in a calcaneofibular articulation.

**Figure 3.**
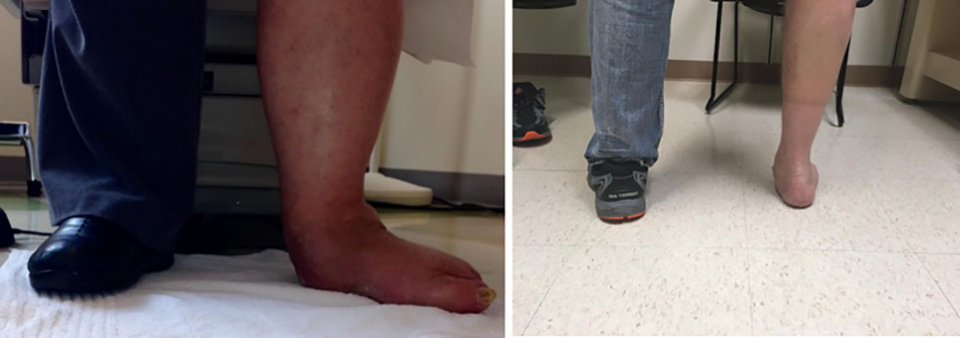
Clinical picture of standing alignment.

Physical examination should be done with both shoes and socks removed. This examination can reveal medial-sided pain due to attenuation and inflammation of the remnant of the tibialis posterior tendon and spring ligament. Lateral pain can be due to calcaneofibular impingement, peroneal tendon irritation, sinus tarsi syndrome, and subtalar arthritic pain (Figure 2). The patient should be viewed from anterior and posterior in a standing position to determine the amount of hindfoot valgus and forefoot abduction that is present (Figure 3). Single-limb heel rise should be assessed. Patients with degeneration of the posterior tibial tendon (stages 2 and 3) will be unable to correct the heel into varus during attempted heel rise. Range of motion of the ankle, subtalar, and talonavicular joint should be checked. The Silverskiold test is performed to differentiate gastrocnemius from tendoachilles contracture. It should be performed with the hindfoot in neutral and the transverse tarsal joints locked. However, in the case of rigid deformity, it is impossible to manually correct hindfoot valgus and abduction through the midfoot, and therefore it becomes impossible to perform an accurate Silverskiold test. Despite the inability to obtain an accurate test, most of these patients will require a gastrocnemius recession or tendoachilles lengthening when the triple arthrodesis is performed. Once the hindfoot is in a neutral position and the transverse tarsal joints are in a corrected position, the patient will likely be unable to attain neutral dorsiflexion without lengthening of 1 or both of the posterior musculotendinous units. Inspection of the peroneal tendons is also useful to investigate for contracture. When the peroneals are contracted, a lengthening or release should be performed to allow for correction of deformity. A Semmes Weinstein monofilament test should be performed to evaluate for neuropathy. Final dorsal and pedal pulses should be palpated with referral for vascular studies when abnormal.

## Radiographic Evaluation

**Figure 4.**
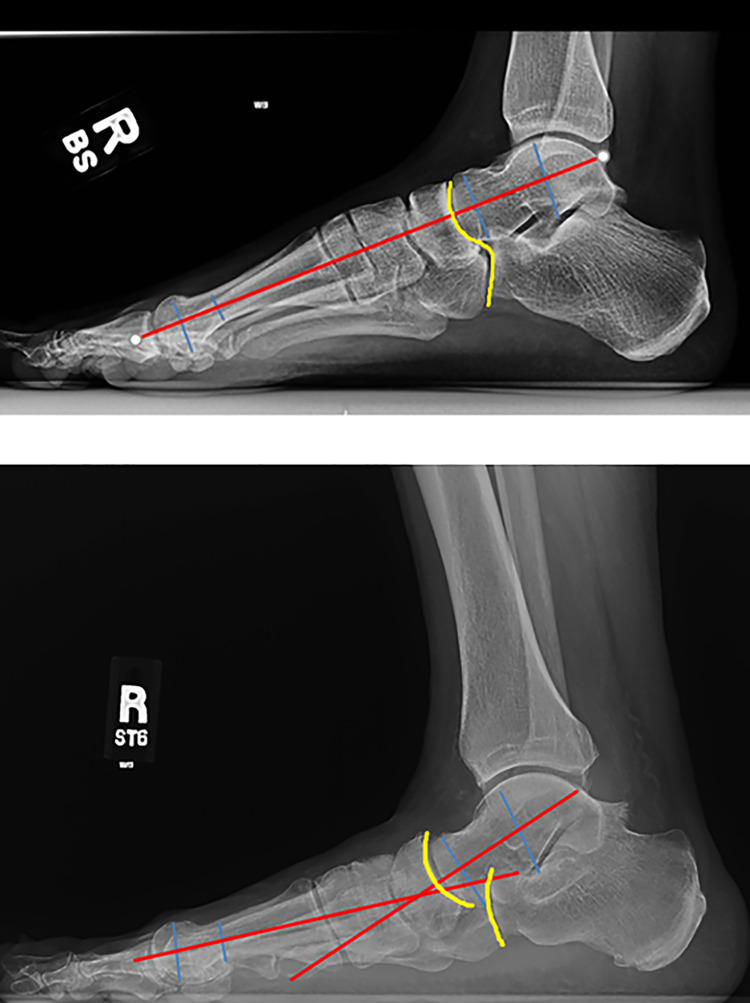
Radiographs of intact Meary’s angle and cyma line as compared to abnormal Meary’s angle and disrupted cyma line in the case of pes planovalgus deformity. The yellow line represents the cyma line. The red and blue lines demonstrate measurement of Meary’s angle by drawing perpendicular lines to the long axis of the talus and the first metatarsal.

**Figure 5.**
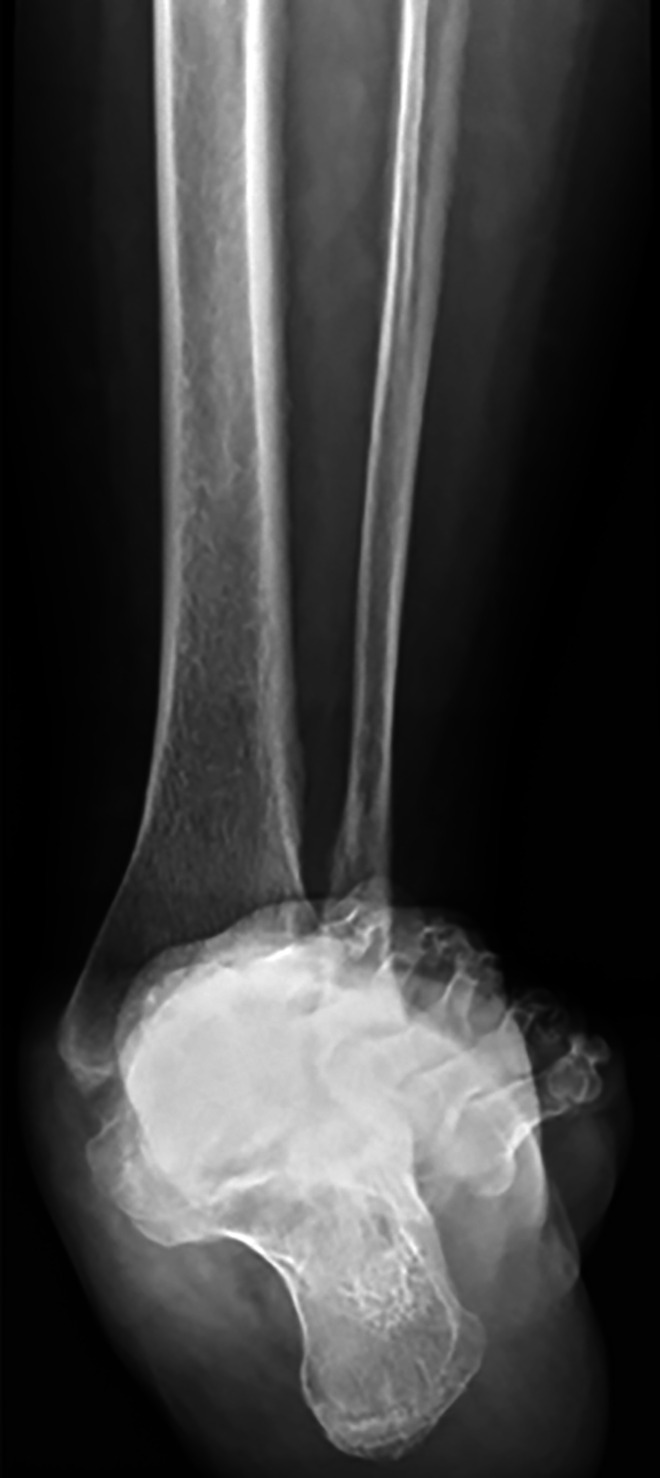
Saltzman hindfoot radiograph.

Weightbearing views of the foot and ankle are essential for preoperative evaluation as well as for operative planning. Evaluation of Meary’s angle on both the anteroposterior and lateral radiographs is useful to quantitate the amount of deformity.^52^ Evaluation of this angle intraoperatively also helps to confirm adequate correction. Other radiographic measurements to evaluate the deformity include the talonavicular coverage angle, calcaneal pitch, the talocalcaneal angle, and the cyma line. The cyma line corresponds to an outline of the Chopart joint and any incongruity of this line indicates pes planus or cavus (Figure 4).^4,69^ The ankle radiographs should be scrutinized for any valgus tilt of the talus that can be indicative of deltoid insufficiency and progression to a stage IV flatfoot deformity. Special views such as the Saltzman view evaluate the position of the calcaneus relative to the tibia (Figure 5). Advanced imaging in the form of a computed tomography (CT) scan is helpful to investigate joint degeneration, coalitions, and osseous cyst formation. If available, a weightbearing CT scan can help to better characterize the 3-dimensional malalignment of the hindfoot and midfoot.^3,64^ The role of magnetic resonance imaging (MRI) in the rigid flatfoot is limited and may be useful only if there is concern for deltoid insufficiency that is not clear on examination or for better defining tibialis posterior tendon pathology.

## Double vs Triple Arthrodesis

Prior to surgery, the decision should be made whether to include the calcaneocuboid joint in the arthrodesis construct. This decision should be made based on physical examination findings such as tenderness over the joint as well as radiographic findings consistent with degenerative changes or subluxation of the joint. When the joint is not included in the final construct, there are reports of an incidental arthrodiastasis occurring through the joint as a result of the correction of forefoot abduction. Those with higher grades of degeneration in the calcaneocuboid joint were shown to radiographically benefit less from the arthrodiastasis.^9^ While this phenomenon is described and observed radiographically, no evidence correlates distraction of the calcaneocuboid joint to improved outcomes.^47,68,83^ Also important to consider is that following fusion of the talonavicular joint, motion at the calcaneocuboid joint is almost nil.^5^ One recent study found significantly higher nonunion rate and significant lower patient outcome scores in those who underwent double arthrodesis vs triple arthrodesis for rigid planovalgus deformity.^13^ The use of a double arthrodesis, however, does not appear to adversely affect the radiographic correction of deformity.^13,23^

## Approach

Multiple approaches to the triple joint complex have been described. These include various lateral approaches combined with a medial incision or an isolated medial approach. Lateral approaches include the Ollier incision and the extended lateral incision. Dangers in both cases include the sural nerve, the peroneus tertius, the extensor digitorum brevis, and the peroneal tendons. The calcaneocuboid joint can be accessed using either approach, as can the lateral aspect of the talonavicular joint, but this is dependent on anatomy, the amount of deformity present, and surgeon experience. Rigid deformity by nature will make joint distraction and visualization more difficult. Despite the concern that lateral approaches will be difficult to close following correction of longstanding deformity, 1 recent study investigated a single lateral approach compared to the standard 2-incision approach to triple arthrodesis. This retrospective review demonstrated no increase in wound complications with a single lateral approach and noted no difference in wound-healing rates or complications while the single lateral approach enjoyed a shorter surgical time.^55^

The medial “utility” incision spans from the medial malleolus to the navicular tuberosity and allows for access to the medial subtalar joint and most of the talonavicular joint. In the case of flatfoot deformity, this access is improved due to talar head uncoverage. The talonavicular joint, however, is notoriously difficult to prepare due to the concave nature of the navicular, which may account for the approximately 6% to 20.4% risk of nonunion at this joint observed across multiple studies.^10,15,45,68^ Appreciation of the depth and “ball-and-socket” nature of the joint is the key to adequate joint preparation. Using a freer elevator, a small joint distractor or a laminar spreader will allow most of the joint to be visualized from the medial side. Alternative medial approaches can be shifted dorsally to allow access to the naviculocuneiform and first tarsometatarsal joint if needed.

A single medial approach is attractive in cases with longstanding rigid deformity because the lateral skin and soft tissues in these patients are contracted. One study investigated patients with lateral skin at risk as a result of rigid pes planovalgus deformity who underwent an isolated medial arthrodesis. They noted no wound complications in their series of 11 patients.^12^ Dangers of the single medial approach should also be taken into account, as this approach risks damage to the blood supply to the talus and inadvertent violation of the anterior fibers of the deltoid.^57^ A cadaveric study investigated injury to the main blood supply to the talus in the case of a single medial approach vs dual approaches. The single medial approach consistently disrupted the main vascular supply while the dual approach did so in varying degrees.^62^ As this was a cadaveric study, it is difficult to correlate the findings to patient outcomes and rates of osteonecrosis following a single vs dual approach to triple arthrodesis. Another important consideration with an isolated medial approach is access to the calcaneocuboid joint. It is reported that anywhere from 36% to 90% of the calcaneocuboid joint can be prepared via the medial approach based on cadaveric studies, but these cadaveric studies did not take deformity into account.^41,62^

**Figure 6.**
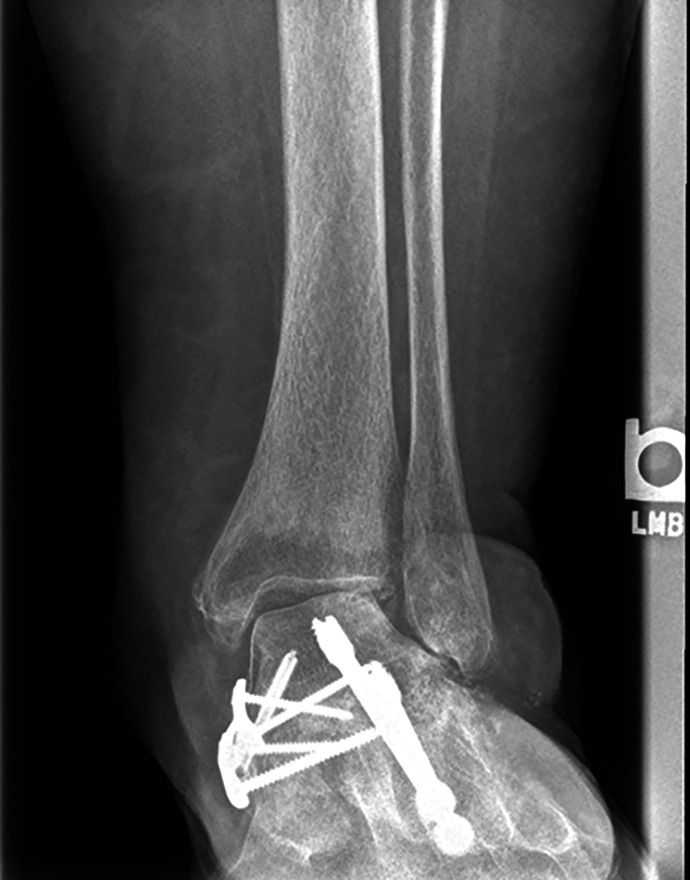
Postsurgical ankle valgus following medial double arthrodesis.

Valgus instability of the ankle can occur following triple or double arthrodesis if the tibiocalcaneal fibers of the superficial deltoid are violated during the medial approach as they contribute to the valgus stability of the ankle (Figure 6).^30^ In 1 study, 2 of 18 patients (11%) who underwent a single medial approach double arthrodesis developed postoperative ankle valgus, whereas another study reported a 27% rate of this complication. The latter study cited more preoperative deformity as a risk factor for postoperative ankle valgus.^1,54^ Undercorrection of hindfoot valgus may contribute to this complication, and a medial displacement calcaneal osteotomy should be performed if an inadequate correction is achieved through the joint.^35^

## Correction of Deformity

**Figure 7.**
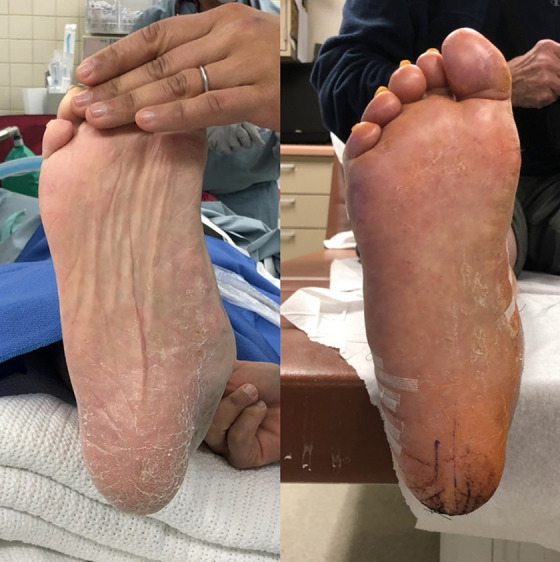
Clinical evaluation of hindfoot position.

**Figure 8.**
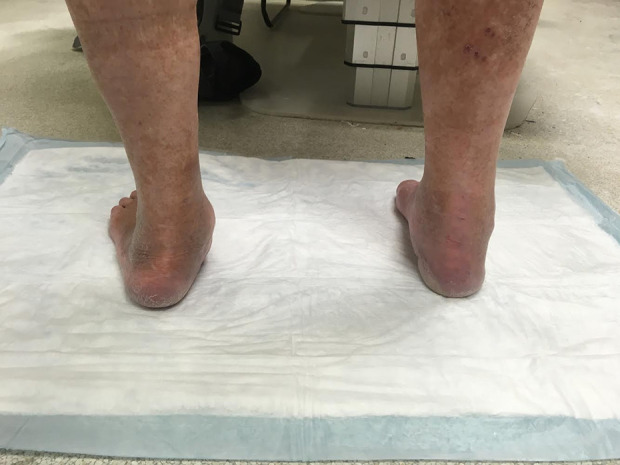
Postoperative foot positioning compared with contralateral foot.

After the triple joint complex has been prepared by cartilage removal and subchondral drilling/burring, the correction is performed. Intraoperative evaluation and reevaluation following correction of deformity can be difficult. A “before-and-after” clinical examination can be helpful as can an intraoperative Saltzman view to look at the hindfoot alignment with respect to the tibia. Prior to placing talonavicular and subtalar screws for final fixation, careful scrutiny of both of these should be performed to prevent under- or overcorrection. The Grice maneuver is a useful intraoperative tool to allow for correction of flatfoot deformity, but it is powerful, and overcorrection is possible. A lamina spreader is placed between the anterior process of the calcaneus and the lateral shoulder of the talus with the aim of lateral and dorsal rotation of the foot around the talus.^35^ This opens the sinus tarsi and corrects the deformity. Once this is achieved, it is provisionally fixed with large K-wires or Steimann pins and evaluated clinically and radiographically prior to placement of definitive fixation. It is difficult to re-create a simulated weightbearing position of the foot intraoperatively, but a flat plate examination can be useful in addition to a fluoroscopic Saltzman view. A clinical evaluation can also be performed, viewing the patient from the foot of the table to ensure appropriate correction (Figure 7). Postoperatively, and once weightbearing is allowed, the patient should be viewed again in a standing position to ensure the forefoot and hindfoot are well aligned (Figure 8).

**Figure 9.**
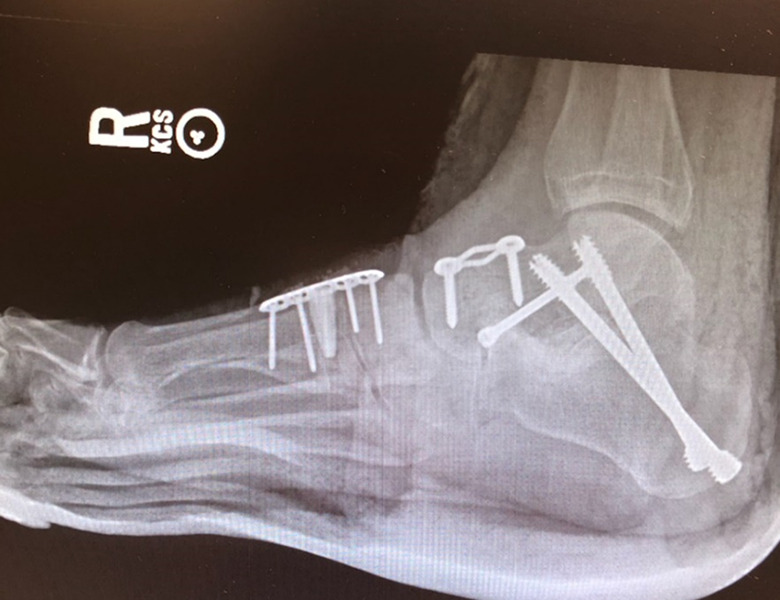
Titanium wedge used to plantarflex the first ray.

An important consideration following correction of the hindfoot is that of residual forefoot varus or supination. When present, this should be addressed with a cotton osteotomy or plantarflexion arthrodesis of the first tarsometatarsal joint. The decision-making process between these procedures is dictated by hypermobility of the medial ray. If hypermobility is present, a first tarsometatarsal joint fusion with or without inclusion of the naviculocuneiform joint is warranted. If hypermobility is not present, a cotton osteotomy can be performed. Precontoured allograft and titanium wedges can be useful to dial in plantarflexion of the ray and avoid shortening of the medial column (Figure 9).

## Fixation

A variety of fixation options are available, including solid and cannulated screws of various sizes, conventional and PEEK plates, and staples. The decision of which to use should be based on the surgeon’s comfort with the available implants. Cannulated fixation allows for provisional fixation using guide wires and can be helpful in a teaching environment by allowing multiple passes for proper positioning. The use of staples is also growing in popularity, and cadaveric studies have shown no strength difference between staples and screws for fixation of the triple joint complex.^53,60^ A recent study retrospectively demonstrated a high union rate (95.7%) in hindfoot and midfoot arthrodesis using a combination screw and nitinol staple construct. A staple-only construct also yielded union rates above 90%.^71^

When using screw fixation of the subtalar joint, more than 1 screw should be used, and placement of the screws in divergent fashion has demonstrated greater torsional stiffness when compared to a single screw and 2 screws placed in parallel fashion in a cadaver study.^14^ The direction of screw insertion, dorsal to plantar (talus to calcaneus) or plantar to dorsal (calcaneus to talus), has also been examined. One study found increased pullout strength with dorsal to plantar screw placement. Interestingly, however, when the plantar to dorsal screws engaged the medial wall of the calcaneus, the pullout strength almost doubled. Complications of dorsal to plantar screw placement include neurovascular injury, insult to talar blood supply, and anterior ankle impingement if the screw is left proud.^33,51^ Talonavicular fixation can be achieved in a number of ways, the most common of which is retrograde screw placement. This method of fixation should be performed cautiously, as 1 cadaveric study found that dorsolateral retrograde screw placement resulted in injury to the deep peroneal nerve, dorsalis pedis, and/or superficial peroneal nerve branch in 5 of 17 specimens.^6^

## Bone Graft

Autograft bone for triple arthrodesis can be harvested from a number of locations, including proximal and distal tibia, calcaneus, and iliac crest. While autograft has been considered the “gold standard” for arthrodesis, donor site morbidity and increased operative time along with the availability and success of orthobiologics have pushed many surgeons toward using allograft. The main disadvantages of allograft include cost, lack of viable cells, and risk of disease transmission. Allograft bone is offered as cancellous chips, structural graft, powders, putties, pastes, and demineralized bone matrices. Some allograft options boast viable cells, with promising results in high-risk foot and ankle reconstructions demonstrated in 1 retrospective study.^73^ Also available are calcium phosphate and sulfate compounds. A number of studies have investigated bone graft substitutes for use in hindfoot fusions, and a few have compared these substitutes to autologous bone with heterogenous results.^27,50^ One prospective study investigated autograft bone vs recombinant human platelet-derived growth factor BB (rhPDGF-BB) mixed with tricalcium phosphate in a foot and ankle fusion group and found a higher rate of radiographic fusion at 36 months postoperatively in the allograft patients vs the autograft group (77% vs 50%).^25^ Bioactive glass is yet another option. When mixed with bone marrow aspirate, 1 retrospective study reported a union rate comparable to that of autograft.^74^

## Adjunct Procedures

In the face of longstanding pes planus, various structures can become contracted. Most common is the tendoachilles or isolated gastrocnemius contracture. An important intraoperative examination, after the hindfoot has been corrected, is the Silverskiold test. This test allows the examiner to differentiate between an isolated gastrocnemius contracture and a gastrocsoleus contracture. Treatment differs as the former can be treated with a gastrocnemius recession whereas the latter should be treated with tendoachilles lengthening, most commonly by a triple cut. A peroneal tendon release may also be needed to correct the deformity, and this can be done through the lateral incision used to prepare the subtalar joint. If a lateral incision is not used, a small retromalleolar incision can be made for peroneal release.

## Postoperative Protocol

After the incisions are carefully closed with suture of choice, a well-padded postoperative splint should be placed in neutral dorsiflexion. Pain control in the form of regional anesthesia, whether that be a single shot or continuous via catheter, can help decrease narcotic pain requirements following the procedure. The patient can be admitted to the hospital or treated as an outpatient, depending on the surgeon’s preference. The patient is seen back at 2 to 3 weeks following the procedure for suture removal and transition into a short-leg, nonweightbearing fiberglass cast. In the case of neuropathic patients, regular cast and wound checks are advisable. The patient is seen again at 6 to 7 weeks, and radiographs are obtained. Due to the expected postoperative swelling in this patient population, there are various protocols aimed at reducing edema via compression dressings. While most of these studies are aimed at postoperative care of traumatic injuries such as ankle fractures, the principles can be applied to the arthrodesis patient. These protocols involve a variety of pneumatic intermittent impulse compression devices to a cotton batting compression dressing placed in the operating room. Volumetric measurements demonstrate a decrease in postoperative swelling with these regimens, and they are generally tolerated well by patients.^29,59,65,72,78^ The patient’s weightbearing can be gradually advanced at that point if the surgeon feels there is enough osseous healing, or the patient can be kept off of the extremity for an additional 3 to 4 weeks. The patient is transitioned into a boot regardless of weightbearing status at 6 weeks to allow for initiation of ankle range of motion with physical therapy assistance. The patient is monitored at regular intervals and progressed as he or she tolerates and as radiographs dictate. CT scan is useful in determining whether a nonunion or delayed union is present as radiographs have been shown to have poor accuracy in quantifying the proportion of joint surface that has achieved fusion.^17,43^ A bone stimulator can be considered at the 3-month mark or beyond if there is questionable progression toward arthrodesis. While most studies have evaluated healing of fracture nonunion, there are some studies investigating union rates using implantable stimulators at the time of arthrodesis surgery in high-risk patients and revision cases.^22,37,48^ A review article in 2006 summarized the clinical evidence of 1 prospective trial and 3 level IV studies aimed at determining the efficacy of bone stimulation in foot and ankle arthrodesis. The authors concluded that electrical bone stimulation may be useful in foot and ankle arthrodesis, but there is insufficient evidence to support its use in the setting of primary arthrodesis but may be useful in long bone delayed unions and nonunions.^44^ A randomized control trial did, however, find that in the setting of primary arthrodesis, pulsed electromagnetic field bone stimulation resulted in decreased time to fusion of the talonavicular and calcaneocuboid joints without a significant difference in time to fusion of the subtalar joint.^24^

## Results and Outcomes

One study reviewed their experience with 42 patients affected by polio 25 years after their triple arthrodesis was performed. Degenerative ankle changes developed in 12 of the 42 (28.5%), and the midfoot developed arthritic changes in 9 of 42 patients (21%).^36^ Another study included even further follow-up of triple arthrodesis patients out to 44 years postprocedure. At average 44-year follow-up, all patients had degenerative changes at the ankle while 33 of the patients had knee or hip pain as well.^66^ Yet another long-term study found that in 30 triples, 6 had undergone ankle fusion at average 21-year follow-up.^84^ Accelerated degeneration of the adjacent joints following arthrodesis in the foot and ankle is a well-described phenomenon as increases in loads across the ipsilateral ankle have been observed following adjacent joint arthrodesis.^7,16,76,86^ Peak pressures at the ankle joint following triple arthrodesis vs selective talonavicular arthrodesis are higher, leading some to advocate selective fusion if possible.^77^ Degenerative change of the ankle appears to be time and age related as a lower rate of arthritis and conversion to ankle fusion is reported at shorter follow-up intervals. In addition, degenerative changes appear sooner when the index procedure is done later in life.^8,31,80^ Despite the presence of progression of radiographic changes, this does not always correlate to pain scores or patient satisfaction with the procedure. Many patients report good outcomes despite accelerated adjacent joint degeneration.^2,31,32,61,75^ Much like arthritis at the calcaneocuboid joint at index procedure, an increased grade of osteoarthritis at the tibiotalar joint when undergoing triple arthrodesis increases the risk of progression postoperatively. Despite this, older adults still report a high rate of satisfaction with the procedure and overall appearance of the foot.^20,32^ The postoperative radiographic alignment of the foot greatly affects patient satisfaction but was shown in 1 study to not affect the progression of degenerative changes about the ankle. Those with neurogenic conditions generally have worse postoperative American Orthopaedic Foot & Ankle Society (AOFAS) scores.^18,46,61^ Functional and gait analyses were investigated in 1 study at an average of 5 years following double or triple arthrodesis. AOFAS scores were rated as good to excellent in most patients. Gait analysis demonstrated decreased plantarflexion and dorsiflexion of the ankle with a significant decrease in power generation through the tibiotalar joint.^7^ Another cadaver model found subtalar arthrodesis to have the most detrimental effect on tibiotalar biomechanics, and additive fusions of the talonavicular and calcaneocuboid joints did not further significantly affect the ankle joint.^39^ It is, however, difficult to apply this cadaver model to actual weightbearing and gait following these procedures. Despite overall positive outcomes following triple arthrodesis, it is important to keep in mind that over- and undercorrection, malunion, and nonunion are concerns and that there is a steep learning curve with regard to correction, appropriate joint preparation, fixation, and soft tissue handling. A recent systematic review including 13 studies in over 481 patients demonstrated a nonunion rate of 6.5% in a variety of joints and wound complication rate of 10%.^82^

## Complications

**Figure 10.**
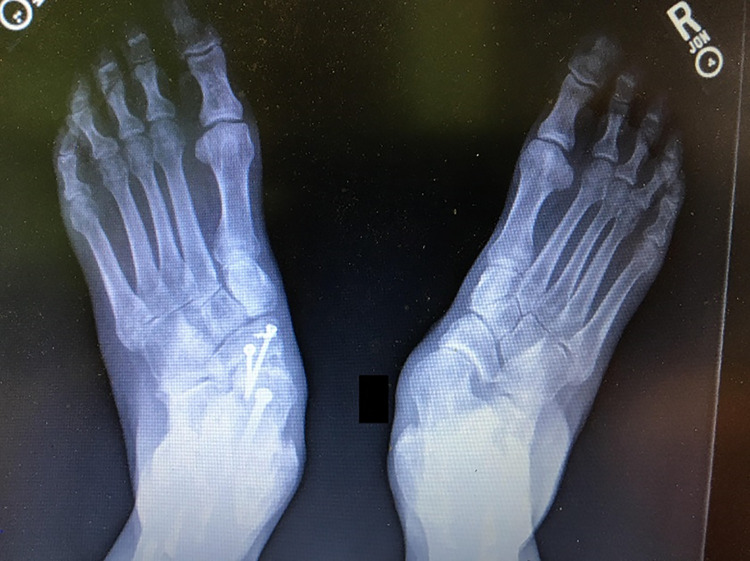
Malpositioned arthrodesis.

The failed triple arthrodesis due to nonunion or malposition is a frustration to the patient and surgeon alike (Figure 10). While the use of internal fixation, improved understanding of anatomy, and bone-grafting techniques have improved the reliability and outcomes of the surgery, the inability to achieve a durable arthrodesis is often multifactorial. The ultimate goal in revision is to avoid soft tissue complications, achieve a solid fusion, and provide the patient with a plantigrade foot that will accommodate orthoses and shoe wear.

Frequently, arthrodesis revision involves operating through an already tenuous soft tissue envelope, removing broken hardware, and/or addressing nonviable bone and the resultant void after debridement. One retrospective study evaluated the failure rates and causes of 302 triple arthrodesis procedures in rheumatoid patients. There were 21 failures (6.8%), of which 66% were malunions and 26% were nonunions. The malunions were most commonly attributable to inadequate correction of deformity or hindfoot malpositioning in either varus or valgus. Revision success rate was 86%.^49^ Another retrospective study investigated 33 patients who underwent revision for triple arthrodesis, using osteotomies and structural graft in some cases for correction of deformity. Clinical and radiographic outcomes improved despite a reoperation rate of 14%.^34^ In the case of malpositioning resulting in rocker bottom deformity, a biplanar midfoot osteotomy can be used to correct abduction and dorsiflexion with positive effects reflected in improved indices of patient satisfaction.^79^ In addition to addressing the primary reason for arthrodesis failure, it is also important to assess whether additional procedures are necessary to address ankle arthritis, forefoot or hindfoot positioning, and/or tendon imbalance.

## Conclusion

Double and triple arthrodesis is a technically demanding but useful procedure to address the deformity and pain of rigid pes planovalgus. Preoperative planning, intraoperative execution, and postoperative rehabilitation are critical to success. It is important for the surgeon to be familiar with the anatomy of the foot, details of the 3-dimensional correction, Arbeitsgemeinschaft für Osteosynthesefragen principles, soft tissue handling, and how to manage complications. Failed procedures can be addressed with revision, but complication rates are higher than those seen in primary procedures.

## Supplemental Material

Supplemental Material, FAO849609-ICMJE - Triple Arthrodesis for Adult-Acquired Flatfoot Deformity Click here for additional data file. Supplemental Material, FAO849609-ICMJE for Triple Arthrodesis for Adult-Acquired Flatfoot Deformity by MAJ Uma E. Erard, MAJ Andrew J. Sheean and Bruce J. Sangeorzan in Foot & Ankle Orthopaedics

## References

[1] Anand P Nunley JA DeOrio JK . Single-incision medial approach for double arthrodesis of hindfoot in posterior tibialis tendon dysfunction. Foot Ankle Int. 2013;34(3):338–344. 23520290 10.1177/1071100712468564

[2] Angus PD Cowell HR . Triple arthrodesis: a critical long-term review. J Bone Joint Surg Br. 1986;68(2):260–265. 3958012 10.1302/0301-620X.68B2.3958012

[3] Apostle KL Coleman NW Sangeorzan BJ . Subtalar joint axis in patients with symptomatic peritalar subluxation compared to normal controls. Foot Ankle Int. 2014;35(11):1153–1158. 25104749 10.1177/1071100714546549

[4] Apostle KL Sangeorzan BJ . Anatomy of the varus foot and ankle. Foot Ankle Clin. 2012;17(1):1–11. 22284548 10.1016/j.fcl.2011.11.001

[5] Astion DJ Deland JT Otis JC Kenneally S . Motion of the hindfoot after simulated arthrodesis. J Bone Joint Surg Am. 1997;79(2):241–246. 9052546 10.2106/00004623-199702000-00012

[6] Atwater LC Aynardi M Melvani R Shon LC Miller SD . Safety of dorsolateral talonavicular joint fixation in modified double arthrodesis: an anatomic study. Current Orthopaedic Practice. 2018;29(3):265–269.

[7] Beischer AD Brodsky JW Pollo FE Peereboom J . Functional outcome and gait analysis after triple or double arthrodesis. Foot Ankle Int. 1999;20(9):545–553. 10509680 10.1177/107110079902000902

[8] Bennett GL Graham CE Mauldin DM . Triple arthrodesis in adults. Foot Ankle. 1991;12(3):138–143. 1686432 10.1177/107110079101200302

[9] Berlet GC Hyer CF Scott RT Galli MM . Medial double arthrodesis with lateral column sparing and arthrodiastasis: a radiographic and medical record review. J Foot Ankle Surg. 2015;54(3):441–444. 25488190 10.1053/j.jfas.2014.10.012

[10] Bibbo C Anderson RB Davis WH . Complications of midfoot and hindfoot arthrodesis. Clin Orthop Relat Res. 2001;391:45–58. 10.1097/00003086-200110000-00007 11603689

[11] Bono JV Jacobs RL . Triple arthrodesis through a single lateral approach: a cadaveric experiment. Foot Ankle. 1992;13(7):408–412. 1427533 10.1177/107110079201300709

[12] Brilhault J . Single medial approach to modified double arthrodesis in rigid flatfoot with lateral deficient skin. Foot Ankle Int. 2009;30(1):21–26. 19176181 10.3113/FAI.2009.0021

[13] Burrus MT Werner BC Carr JB Perumal V Park JS . Increased failure rate of modified double arthrodesis compared with triple arthrodesis for rigid pes planovalgus. J Foot Ankle Surg. 2016;55(6):1169–1174. 27614822 10.1053/j.jfas.2016.07.001

[14] Chuckpaiwong B Easley ME Glisson RR . Screw placement in subtalar arthrodesis: a biomechanical study. Foot Ankle Int. 2009;30(2):133–141. 19254508 10.3113/FAI-2009-0133

[15] Clain MR Baxter DE . Simultaneous calcaneocuboid and talonavicular fusion. Long-term follow-up study. J Bone Joint Surg Br. 1994;76(1):133–136. 8300657

[16] Coester LM Saltzman CL Leupold J Pontarelli W . Long-term results following ankle arthrodesis for post-traumatic arthritis. J Bone Joint Surg Am. 2001;83(2):219–228. 11216683 10.2106/00004623-200102000-00009

[17] Coughlin MJ Grimes JS Traughber PD Jones CP . Comparison of radiographs and CT scans in the prospective evaluation of the fusion of hindfoot arthrodesis. Foot Ankle Int. 2006;27(10):780–787. 17054877 10.1177/107110070602701004

[18] Daglar B Deveci A Delialioglu OM et al. Results of triple arthrodesis: effect of primary etiology. J Orthop Sci. 2008;13(4):341–347. 18696193 10.1007/s00776-008-1243-5

[19] Daniels TR Smith JW Ross TI . Varus malalignment of the talar neck: its effect on the position of the foot and on subtalar motion. J Bone Joint Surg Am. 1996;78(10):1559–1567. 8876585 10.2106/00004623-199610000-00015

[20] de Heus JA Marti RK Besselaar PP Albers GH . The influence of subtalar and triple arthrodesis on the tibiotalar joint: a long-term follow-up study. J Bone Joint Surg Br. 1997;79(4):644–647. 9250757 10.1302/0301-620x.79b4.7194

[21] Deland JT . Adult-acquired flatfoot deformity. J Am Acad Orthop Surg. 2008;16(7):399–406. 18611997 10.5435/00124635-200807000-00005

[22] DeVries JG Berlet GC Hyer CF . Union rate of tibiotalocalcaneal nails with internal or external bone stimulation. Foot Ankle Int. 2012;33(11):969–978. 23131443 10.3113/FAI.2012.0969

[23] DeVries JG Scharer B . Hindfoot deformity corrected with double versus triple arthrodesis: radiographic comparison. J Foot Ankle Surg. 2015;54(3):424–427. 25432460 10.1053/j.jfas.2014.09.020

[24] Dhawan SK Conti SF Towers J Abidi NA Vogt M . The effect of pulsed electromagnetic fields on hindfoot arthrodesis: a prospective study. J Foot Ankle Surg. 2004;43(2):93–96. 15057855 10.1053/j.jfas.2004.01.007

[25] Digiovanni CW Baumhauer J Lin SS et al. Prospective, randomized, multi-center feasibility trial of rhPDGF-BB versus autologous bone graft in a foot and ankle fusion model. Foot Ankle Int. 2011;32(4):344–354. 21733435 10.3113/FAI.2011.0344

[26] Dix B Grant-McDonald L Catanzariti A Saltrick K . Preoperative anemia in hindfoot and ankle arthrodesis. Foot Ankle Specialist. 2017;10(2):109–115. 27613815 10.1177/1938640016666921

[27] Dolan CM Henning JA Anderson JG Bohay DR Kornmesser MJ Endres TJ . Randomized prospective study comparing tri-cortical iliac crest autograft to allograft in the lateral column lengthening component for operative correction of adult acquired flatfoot deformity. Foot Ankle Int. 2007;28(1):8–12. 17257531 10.3113/FAI.2007.0002

[28] Duncan JW Lovell WW . Hoke triple arthrodesis. J Bone Joint Surg Am. 1978;60(6):795–798. 701313

[29] Eagan M SooHoo NF Cracchiolo A III . A cotton batting compression dressing and fiberglass cast used safely in the immediate postoperative period after hindfoot or ankle surgery. Foot Ankle Int. 2006;27(9):706–710. 17038282 10.1177/107110070602700909

[30] Earll M Wayne J Brodrick C Vokshoor A Adelaar R . Contribution of the deltoid ligament to ankle joint contact characteristics: a cadaver study. Foot Ankle Int. 1996;17(6):317–324. 8791077 10.1177/107110079601700604

[31] Ebalard M Le Henaff G Sigonney G et al. Risk of osteoarthritis secondary to partial or total arthrodesis of the subtalar and midtarsal joints after a minimum follow-up of 10 years. Orthop Traumatol Surg Res. 2014;100(4(suppl):S231–S237. 24726756 10.1016/j.otsr.2014.03.003

[32] Graves SC Mann RA Graves KO . Triple arthrodesis in older adults: results after long-term follow-up. J Bone Joint Surg Am. 1993;75(3):355–362. 8444913 10.2106/00004623-199303000-00006

[33] Greisberg J Sangeorzan B . Hindfoot arthrodesis. J Am Acad Orthop Surg. 2007;15(1):65–71. 17213383 10.5435/00124635-200701000-00007

[34] Haddad SL Myerson MS Pell RF Schon LC . Clinical and radiographic outcome of revision surgery for failed triple arthrodesis. Foot Ankle Int. 1997;18(8):489–499. 9278743 10.1177/107110079701800806

[35] Hansen ST . Functional Reconstruction of the Foot and Ankle. Philadelphia, PA: Lippincott Williams & Wilkins; 2000.

[36] Haritidis JH Kirkos JM Provellegios SM Zachos AD . Long-term results of triple arthrodesis: 42 cases followed for 25 years. Foot Ankle Int. 1994;15(10):548–551. 7834062 10.1177/107110079401501005

[37] Hockenbury RT Gruttadauria M McKinney I . Use of implantable bone growth stimulation in Charcot ankle arthrodesis. Foot Ankle Int. 2007;28(9):971–976. 17880870 10.3113/FAI.2007.0971

[38] Hoke M . An operation for stabilizing paralytic feet. Am J Orthop Surg. 1921;3:494–507.

[39] Hutchinson ID Baxter JR Gilbert S et al. how do hindfoot fusions affect ankle biomechanics: a cadaver model. Clin Orthop Relat Res. 2016;474(4):1008–1016. 26689585 10.1007/s11999-015-4671-5 PMC4773330

[40] Ishikawa SN Murphy GA Richardson EG . The effect of cigarette smoking on hindfoot fusions. Foot Ankle Int. 2002;23(11):996–998. 12449402 10.1177/107110070202301104

[41] Jeng CL Tankson CJ Myerson MS . The single medial approach to triple arthrodesis: a cadaver study. Foot Ankle Int. 2006;27(12):1122–1125. 17207442

[42] Johnson KA Strom DE . Tibialis posterior tendon dysfunction. Clin Orthop Relat Res. 1989;(239):196–206. 2912622

[43] Jones CP Coughlin MJ Shurnas PS . Prospective CT scan evaluation of hindfoot nonunions treated with revision surgery and low-intensity ultrasound stimulation. Foot Ankle Int. 2006;27(4):229–235. 16624210 10.1177/107110070602700401

[44] Kesani AK Gandhi A Lin SS . Electrical bone stimulation devices in foot and ankle surgery: types of devices, scientific basis, and clinical indications for their use. Foot Ankle Int. 2006;27(2):148–156. 16487472 10.1177/107110070602700216

[45] Klassen LJ Shi E Weinraub GM Liu J . Comparative nonunion rates in triple arthrodesis. J Foot Ankle Surg. 2018;57(6):1154–1156. 30253967 10.1053/j.jfas.2018.06.006

[46] Klerken T Kosse NM Aarts CAM Louwerens JWK . Long-term results after triple arthrodesis: influence of alignment on ankle osteoarthritis and clinical outcome. Foot Ankle Surg. 2019;25(2):247–250. 29409183 10.1016/j.fas.2017.11.003

[47] Knupp M Schuh R Stufkens SA Bolliger L Hintermann B . Subtalar and talonavicular arthrodesis through a single medial approach for the correction of severe planovalgus deformity. J Bone Joint Surg Br. 2009;91(5):612–615. 19407294 10.1302/0301-620X.91B5.21727

[48] Lau JT Stamatis ED Myerson MS Schon LC . Implantable direct-current bone stimulators in high-risk and revision foot and ankle surgery: a retrospective analysis with outcome assessment. Am J Orthop. 2007;36(7):354–357. 17694182

[49] Maenpaa H Lehto MU Belt EA . What went wrong in triple arthrodesis? An analysis of failures in 21 patients. Clin Orthop Relat Res. 2001;(391):218–223. 11603672

[50] McGarvey WC Braly WG . Bone graft in hindfoot arthrodesis: allograft vs autograft. Orthopedics. 1996;19(5):389–394. 8727332 10.3928/0147-7447-19960501-08

[51] McGlamry MC Robitaille MF . Analysis of screw pullout strength: a function of screw orientation in subtalar joint arthrodesis. J Foot Ankle Surg. 2004;43(5):277–284. 15480401 10.1053/j.jfas.2004.07.009

[52] Meary R. Le pied creux essentiel: Étude anatomo-clinique. Rev Chir Orthop. 1967;53(5):389–410. 4292742

[53] Meyer MS Alvarez BE Njus GO Bennett GL . Triple arthrodesis: a biomechanical evaluation of screw versus staple fixation. Foot Ankle Int. 1996;17(12):764–767. 8973900 10.1177/107110079601701209

[54] Miniaci-Coxhead SL Weisenthal B Ketz JP Flemister AS . Incidence and radiographic predictors of valgus tibiotalar tilt after hindfoot fusion. Foot Ankle Int. 2017;38(5):519–525. 28142248 10.1177/1071100717690439

[55] Moore BE Wingert NC Irgit KS Gaffney CJ Cush GJ . Single-incision lateral approach for triple arthrodesis. Foot Ankle Int. 2014;35(9):896–902. 25005551 10.1177/1071100714539658

[56] Moore KR Howell MA Saltrick KR Catanzariti AR . Risk factors associated with nonunion after elective foot and ankle reconstruction: a case-control study. J Foot Ankle Surg. 2017;56(3):457–462. 28476384 10.1053/j.jfas.2017.01.011

[57] Mulfinger GL Trueta J . The blood supply of the talus. J Bone Joint Surg Br. 1970;52(1):160–167. 5436202

[58] Myerson MS . Adult acquired flatfoot deformity: treatment of dysfunction of the posterior tibial tendon. Instr Course Lect. 1997;46:393–405. 9143981

[59] Myerson MS Henderson MR . Clinical applications of a pneumatic intermittent impulse compression device after trauma and major surgery to the foot and ankle. Foot Ankle. 1993;14(4):198–203. 8103031 10.1177/107110079301400404

[60] Payette CR Sage RA Gonzalez JV Sartori M Patwardhan A Vrbos L . Triple arthrodesis stabilization: a quantitative analysis of screw versus staple fixation in fresh cadaveric matched-pair specimens. J Foot Ankle Surg. 1998;37(6):472–480. 9879042 10.1016/s1067-2516(98)80024-0

[61] Pell RF Myerson MS Schon LC . Clinical outcome after primary triple arthrodesis. J Bone Joint Surg Am. 2000;82(1):47–57. 10653083 10.2106/00004623-200001000-00006

[62] Phisitkul P Haugsdal J Vaseenon T Pizzimenti MA . Vascular disruption of the talus: comparison of two approaches for triple arthrodesis. Foot Ankle Int. 2013;34(4):568–574. 23407016 10.1177/1071100713479318

[63] Pinney SJ Lin SS . Current concept review: acquired adult flatfoot deformity. Foot Ankle Int. 2006;27(1):66–75. 16442033 10.1177/107110070602700113

[64] Probasco W Haleem AM Yu J Sangeorzan BJ Deland JT Ellis SJ . Assessment of coronal plane subtalar joint alignment in peritalar subluxation via weight-bearing multiplanar imaging. Foot Ankle Int. 2015;36(3):302–309. 25380775 10.1177/1071100714557861

[65] Rohner-Spengler M Frotzler A Honigmann P Babst R . Effective treatment of posttraumatic and postoperative edema in patients with ankle and hindfoot fractures: a randomized controlled trial comparing multilayer compression therapy and intermittent impulse compression with the standard treatment with ice. J Bone Joint Surg Am. 2014;96(15):1263–1271. 25100773 10.2106/JBJS.K.00939

[66] Saltzman CL Fehrle MJ Cooper RR Spencer EC Ponseti IV . Triple arthrodesis: twenty-five and forty-four-year average follow-up of the same patients. J Bone Joint Surg Am. 1999;81(10):1391–1402. 10535589

[67] Samilson RL Dillin W . Cavus, cavovarus, and calcaneocavus: an update. Clin Orthop Relat Res. 1983;177:125–132. 6861385

[68] Sammarco VJ Magur EG Sammarco GJ Bagwe MR . Arthrodesis of the subtalar and talonavicular joints for correction of symptomatic hindfoot malalignment. Foot Ankle Int. 2006;27(9):661–666. 17038274 10.1177/107110070602700901

[69] Sangeorzan BJ Mosca V Hansen ST Jr . Effect of calcaneal lengthening on relationships among the hindfoot, midfoot, and forefoot. Foot Ankle. 1993;14(3):136–141. 8491427 10.1177/107110079301400305

[70] Sangeorzan BJ Smith D Veith R Hansen ST Jr . Triple arthrodesis using internal fixation in treatment of adult foot disorders. Clin Orthop Relat Res. 1993;294:299–307. 8358933

[71] Schipper ON Ford SE Moody PW Van Doren B Ellington JK . Radiographic results of nitinol compression staples for hindfoot and midfoot arthrodeses. Foot Ankle Int. 2018;39(2):172–179. 29073772 10.1177/1071100717737740

[72] Schipper ON Hsu AR Haddad SL . Reduction in wound complications after total ankle arthroplasty using a compression wrap protocol. Foot Ankle Int. 2015;36(12):1448–1454. 26231196 10.1177/1071100715597437

[73] Scott RT Hyer CF . Role of cellular allograft containing mesenchymal stem cells in high-risk foot and ankle reconstructions. J Foot Ankle Surg. 2013;52(1):32–35. 23102874 10.1053/j.jfas.2012.09.004

[74] Shi E Carter R Weinraub GM . Outcomes of hindfoot arthrodesis supplemented with bioactive glass and bone marrow aspirate: a retrospective radiographic study. J Foot Ankle Surg. 2019;58(1):2–5. 30316643 10.1053/j.jfas.2018.03.048

[75] Smith RW Shen W Dewitt S Reischl SF . Triple arthrodesis in adults with non-paralytic disease: a minimum ten-year follow-up study. J Bone Joint Surg Am. 2004;86(12):2707–2713. 15590857 10.2106/00004623-200412000-00018

[76] Sponseller PD McBeath AA Perpich M . Hip arthrodesis in young patients: a long-term follow-up study. J Bone Joint Surg Am. 1984;66(6):853–859. 6234319 10.2106/00004623-198466060-00005

[77] Suckel A Muller O Herberts T Langenstein P Reize P Wulker N . Talonavicular arthrodesis or triple arthrodesis: peak pressure in the adjacent joints measured in 8 cadaver specimens. Acta Orthop. 2007;78(5):592–597. 17966017 10.1080/17453670710014275

[78] Thordarson DB Ghalambor N Perlman M . Intermittent pneumatic pedal compression and edema resolution after acute ankle fracture: a prospective, randomized study. Foot Ankle Int. 1997;18(6):347–350. 9208293 10.1177/107110079701800607

[79] Toolan BC . Revision of failed triple arthrodesis with an opening-closing wedge osteotomy of the midfoot. Foot Ankle Int. 2004;25(7):456–461. 15319102 10.1177/107110070402500703

[80] Trehan SK Ihekweazu UN Root L . Long-term outcomes of triple arthrodesis in cerebral palsy patients. J Pediatr Orthop. 2015;35(7):751–755. 25393571 10.1097/BPO.0000000000000361

[81] Ryerson E . The classic: arthrodesing operations on the feet. Clin Orthop Relat Res. 1977;122:4–9. 319934

[82] Walker R Francis R Ajuied A . Death of the triple arthrodesis? Orthop Trauma. 2015;29(5):324–333.

[83] Weinraub GM Schuberth JM Lee M et al. Isolated medial incisional approach to subtalar and talonavicular arthrodesis. J Foot Ankle Surg. 2010;49(4):326–330. 20610201 10.1053/j.jfas.2010.04.015

[84] Wetmore RS Drennan JC . Long-term results of triple arthrodesis in Charcot-Marie-Tooth disease. J Bone Joint Surg Am. 1989;71(3):417–422. 2925716

[85] Whitman R . Further observations on the treatment of paralytic talipes calcaneus by astralgectomy and backward displacement of the foot. Ann Surg. 1908;47(2):264–273. 17862117 10.1097/00000658-190802000-00009 PMC1414510

[86] Wu WL Huang PJ Lin CJ Chen WY Huang KF Cheng YM . Lower extremity kinematics and kinetics during level walking and stair climbing in subjects with triple arthrodesis or subtalar fusion. Gait Posture. 2005;21(3):263–270. 15760741 10.1016/j.gaitpost.2004.02.001

[87] Wukich DK Crim BE Frykberg RG Rosario BL . Neuropathy and poorly controlled diabetes increase the rate of surgical site infection after foot and ankle surgery. J Bone Joint Surg Am. 2014;96(10):832–839. 24875024 10.2106/JBJS.L.01302 PMC4018772

